# Mechanism of Self-Assembled Cubic InGaN/GaN Quantum Well Formation in Metal-Modulated Molecular Beam Epitaxy

**DOI:** 10.1021/acs.cgd.5c00202

**Published:** 2025-05-17

**Authors:** Mario F. Zscherp, Silas A. Jentsch, Vitalii Lider, Matthew Chia, Andreas Beyer, Anja Henss, Donat J. As, Kerstin Volz, Sangam Chatterjee, Jörg Schörmann

**Affiliations:** † Institute of Experimental Physics I and Center for Materials Research, 9175 Justus Liebig University Giessen, Heinrich-Buff-Ring 16, Giessen 35392, Germany; ‡ Materials Science Center and Faculty of Physics, 9377 Philipps-University Marburg, Hans-Meerwein-Strasse 6, Marburg 35032, Germany; § Department of Physics, University of Cambridge, Cambridge CB2 1TN, United Kingdom; ∥ Department of Physics, Paderborn University, Warburger Strasse 100, Paderborn 33098, Germany

## Abstract

Alternating metal-modulated molecular beam epitaxy enables the growth of both self-assembled c-InGaN/GaN quantum wells and fully alloyed c-InGaN layers. *In situ* reflection high-energy electron diffraction (RHEED) analysis coupled with *ex situ* structural characterization investigates the growth mechanism and prerequisites for the self-assembled c-InGaN quantum well formation. The data reveal that indium accumulates without incorporating into the underlying c-GaN layer during an indium deposition step. However, the accumulated indium forms c-InGaN during a subsequent GaN growth step consistent with vertical cation segregation. Furthermore, X-ray diffraction, time-of-flight secondary ion mass spectrometry depth profiles, and scanning transmission electron microscopy imaging show homogeneous and well-defined c-InGaN layers. The presented growth mechanism requires high substrate temperatures and gallium fluxes. Still, limit testing suggests that indium contents of up to 37% are feasible. This encourages the implementation of metal-modulated grown c-InGaN in red light-emitting devices. Furthermore, combining RHEED operando diagnostics and a precise understanding of the growth mechanism is vital for progressing toward automated growth of complex heterostructures.

## Graphical abstract

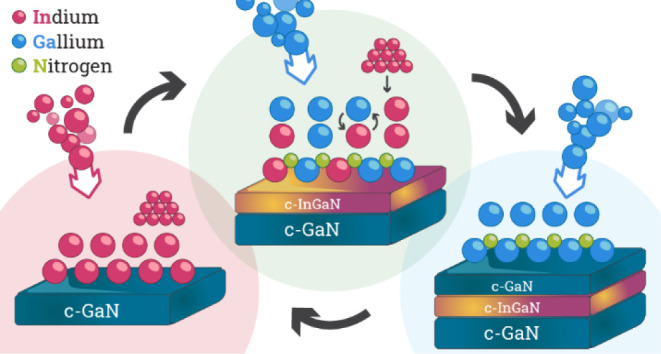

## Motivation and Introduction

The realization of sub 5-μm diameter efficient micro-light-emitting diodes (μ-LED’s) promises to unlock the full potential of augmented and virtual reality (AR and VR) through active displays. Such devices are readily available in blue and green, but the low efficiency of red μ-LEDs remains a bottleneck for the widespread implementation of μ-LEDs. All conventional compounds of inorganic solid-state lighting face major challenges.^1,2^ AlInGaP is the state-of-the-art active material for red LEDs,^3,4^ but its efficiency drops drastically when downscaled^5–7^ to a level no longer suitable; also, covering shorter wavelengths is challenging. Wurtzite InGaN provides very efficient blue light emission.^8–10^ Its emission can cover the entire visible spectrum^11,12^ which is advantageous for the fabrication of RGB μ-LEDs from a single material system. Additionally, reducing the size of wz-InGaN devices is less detrimental due to a lower surface recombination velocity compared to AlInGaP.^13,14^ However, longer emission wavelengths require a higher indium content, which decreases their efficiency due to strong internal fields and high defect densities.^15–19^

Cubic InGaN in the metastable zinc-blende phase has recently reemerged as a promising choice for active material in light emitters.^20–23^ The absence of macroscopic internal polarization fields^24–27^ and red light emission at lower indium contents^21,23^ compared to wz-InGaN are desirable properties for LED applications. Recent simulations suggest a wide single quantum well without an electron blocking layer as the ideal LED design for c-InGaN.^28^ This requires the growth of c-InGaN/GaN quantum well structures with precise control of layer thicknesses, compositions, and interface roughness. The metastability of the cubic phase and the differences in growth conditions, such as the large differences in growth temperatures between c-In*_x_* Ga_1–*x*_N with *x*(In) > 0.2 and c-GaN, pose significant challenges. Metal-modulated epitaxy (MME) enables efficient quantum structure growth. Supplying gallium and indium alternately can yield ternary InGaN quantum wells even though the two group III elements are never supplied simultaneously.^29–31^ Adjusting the duration of the shutter intervals allows seamless tuning between self-assembled c-InGaN/GaN quantum wells and fully alloyed c-InGaN layers.^31^

In this work, we propose the underlying physical mechanism enabling the self-assembled growth of cubic In*_x_* Ga_1–*x*_N/GaN quantum wells. We utilize *in situ* observations during actual growth (“operando”) and external, *ex situ* characterization of specifically designed sample series to unambiguously identify the optimal conditions for bulk c-InGaN and InGaN/GaN MQW formation: during an indium deposition step, indium accumulates without incorporating into the underlying c-GaN layer but forms c-InGaN during a subsequent GaN growth step. These observations are consistent with vertical cation segregation and allow for assessing the intrinsic limitations of metal-modulated growth of c-InGaN/GaN quantum wells. Furthermore, the correlation of in-depth structural analysis with reflection high-energy electron diffraction (RHEED) analysis during growth provides operando diagnostics suitable for predicting structure formation, material properties, and interface quality. These correlations and the understanding of the growth mechanism are essential steps toward implementing machine learning and artificial intelligence approaches in future quantum structure growth.

## Experimental Section

Cubic InGaN/GaN multilayers are grown by metal-modulated plasma-assisted molecular beam epitaxy (PAMBE). The multilayers are deposited on approximately 600 nm thick c-GaN templates, which are grown using a c-AlN buffer^32^ on commercial 3C-SiC/Si (001) substrates (NovaSiC). All samples are grown in a Riber Compact12 chamber using standard effusion cells for the group III elements and an Oxford Applied Research HD25 radio-frequency nitrogen plasma source. RHEED monitors the growth surface during the growth procedure. The metal-modulated growth sequence yields the desired InGaN/GaN multilayers. In this process, the indium and gallium sources are shuttered alternately, while the nitrogen shutter remains constantly open. A brief growth interruption of 5 s in the metal supply prevents intermixing of Ga and In in the gas phase. The beam equivalent pressures (BEP) of gallium and indium vary between 1.3 × 10^–7^ and 2.2 × 10^–7^ mbar and 1.9 × 10^–7^ and 2.9 × 10^–7^ mbar, respectively. The growth temperature of the samples varies from 550 to 650 °C, as measured by a pyrometer.

Reciprocal space maps (RSMs) of the (002) and (−1–13) reflections were measured by high-resolution X-ray diffraction (HRXRD) on a Rigaku SmartLab diffractometer equipped with a 9 kW rotating Cu anode coupled to a HyPix-3000 high-energy-resolution 2D detector.

Time-of-flight secondary ion mass spectrometry (ToF-SIMS) investigations were conducted using an M6plus instrument (IONTOF GmbH) equipped with a 30 kV Bi liquid metal ion gun (LMIG) for analysis and a dual source column (DSC) for depth profiling. Depth profiles were recorded in noninterlaced mode with Cs^+^ as the sputter species at an energy of 500 eV and with Bi^+^ ions for analysis. Each analysis frame was followed by 4 sputter frames after a pause of 0.5 s. The sputter current was 40.21 nA. The LMIG current was 1.13 pA with a cycle time of 100 μs. The size of the sputter crater was 300 × 300 μm^2^, and the analysis field was 75 × 75 μm^2^ with 128 × 128 pixels. Measurements were performed in negative polarity in the spectrometry mode of the LMIG and in the all-purpose mode of the analyzer, achieving a mass resolution of *m*/Δ*m* > 17000 for *m*/*z* = 114.9 (In^–^). An electron flood gun was used for neutralization. The spectra were calibrated to C^–^, C2^–^, and C3^–^, and the data were evaluated with SurfaceLab 7.4 (IONTOF GmbH).

The scanning transmission electron microscopy (STEM) measurements were performed using a double-aberration-corrected JEOL JEM-2200FS microscope with an operating voltage of 200 kV and an annular dark-field (ADF) detector. The high-angle ADF (HAADF) imaging mode was used, providing the so-called Z-contrast.^33^ Electron-transparent cross-section lamellas with thicknesses of a few tens of nanometers were prepared using focused ion beam.

SEM images of the thin films’ cross-sections were acquired with a Zeiss Merlin scanning electron microscope operated at an acceleration voltage of 5 kV.

## Results and Discussion

The *in situ* surface analysis by RHEED provides insights into the resulting structure, i.e., the formation of fully alloyed c-InGaN layers or self-assembled c-InGaN/GaN multi-quantum wells. This wide range of target structures is accessible by alternatingly opening the gallium and indium shutters while continuously supplying nitrogen.^31^ This extended scope becomes possible by modifying the established procedures of metal-modulated epitaxy of ternary nitrides, which commonly open and close the metal shutters simultaneously to oscillate between metal-rich and nitrogen-rich growth conditions.^34–39^ The detailed presentation of the *ex situ* diagnostic results and the inherent limitations of the proposed growth scheme follows the in-depth discussion of the operando diagnostics.

The intensity of the central [110] RHEED streak shown in Figure 1 (left) probes the individual steps of the metal-modulated growth sequence for an exemplary growth of c-InGaN on a c-GaN template (see Figure S1). Step [1] corresponds to the simultaneous indium and nitrogen supply for *t*_In+N_ = 280 s. The RHEED intensity decreases rapidly during the first 10 s after opening the In shutter. Subsequently, the intensity continues to slowly decrease for the remaining duration of this step. This decrease corresponds to the accumulation of indium on the surface. The initial, more rapid decrease indicates the formation of a first closed layer, after which nanodroplets of In form. The set growth temperature (*T*_growth_ ≈ 645 °C) exceeds the decomposition temperature of InN (*T*_InN_ ≈ 500–550 °C).^40,41^ Consistently, no indications of InN formation are found either during growth or in the extensive postgrowth sample analyses.

**Figure 1:**
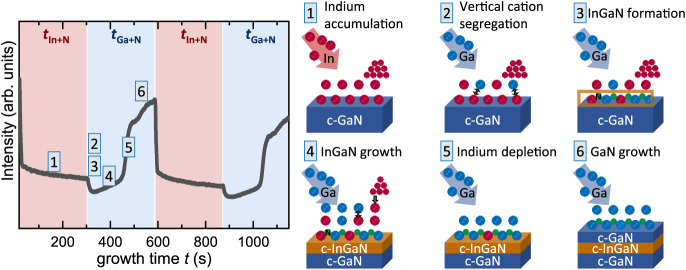
*In situ* RHEED intensity measurements monitor the individual phases of metal-modulated growth. RHEED shows [1] decreasing intensity due to indium accumulation, [2–4] increasing intensity during c-InGaN growth, [5] rapid increase once the indium reservoir is depleted, and [6] high intensity during c-GaN growth.

The brief growth interruption caused by closing the indium shutter prevents intermixing in the gas phase. Opening the gallium shutter for *t*_Ga+N_ = 280 s initializes Ga deposition on the indium-covered surface. Successively, the larger indium atoms segregate toward the surface by exchanging positions with gallium atoms [2]; similar processes often occur in ternary alloys^42,43^ and have recently been modeled to explain self-assembled nanostructures in wurtzite AlGaN.^44^ This vertical cation segregation gradually changes the composition of the metal layer at the crystal interface, transitioning from indium-rich to gallium-rich. The continuously supplied nitrogen diffuses through the metal layer but cannot adsorb onto the metal-saturated crystal surface.^45^ Indium, gallium, and nitrogen eventually form solid c-In*_x_* Ga_1–*x*_N [3] once the indium–gallium ratio reaches an indium content *x*(In) that is thermodynamically stable at this growth temperature.^23^ This mechanism results in a steady state of c-InGaN forming at the crystal–adlayer interface, with the nanodroplets releasing indium into the adlayer, while the effusion cell supplies gallium. Note that the growth of high-quality c-In*_x_* Ga_1–*x*_N with *x*(In) > 0.2 requires precise control of the gallium flux. The flux must be sufficiently high to sustain the adlayer, enabling the indium from the nanodroplets to diffuse on the one hand, while excessive gallium reduces *x*(In)^46,47^ on the other hand due to the preferential incorporation of gallium versus indium.

The growth of c-InGaN gradually consuming the indium nanodroplets causes a slow increase of the RHEED intensity [4]. The depletion of the indium reservoir ends stage [4], rapidly increasing the RHEED intensity as gallium and nitrogen form binary c-GaN [5]. The RHEED intensity remains high during this period [6] until the gallium shutter is closed and the indium shutter is opened again.

Overall, operando RHEED analysis consistently and precisely reveals the growth of both c-InGaN and c-GaN. Understanding the mechanism and identifying the correlation between RHEED data, growth mechanisms, and structural data allow for future implementations of machine learning procedures and reference datasets for training suitable algorithms. These then enable automated fine-tuning of the growth conditions to achieve desired individual layer thicknesses in MQWs or even grow fully alloyed c-InGaN layers^31^ by stopping the gallium supply immediately after consuming all indium droplets.

*Ex situ* characterization using XRD, ToF-SIMS, and STEM investigates the precise conditions leading to c-InGaN formation, verifying the interpretation of the *in situ* RHEED measurements. Figure 2 compares reciprocal space maps (RSMs) centered at the c-GaN (002) reflection of two exemplary samples, each featuring only one indium deposition step (*t*_In+N_). Figure 2a shows the data for the as-grown sample. The X-ray diffraction (XRD) pattern reveals the presence of metallic indium but shows no indication of c-InGaN or c-InN. The latter would occur at the position of the red circle in Figure 2a. These data lead to the assessment that metallic indium accumulates on the surface during *t*_In+N_ without forming c-InGaN.

**Figure 2:**
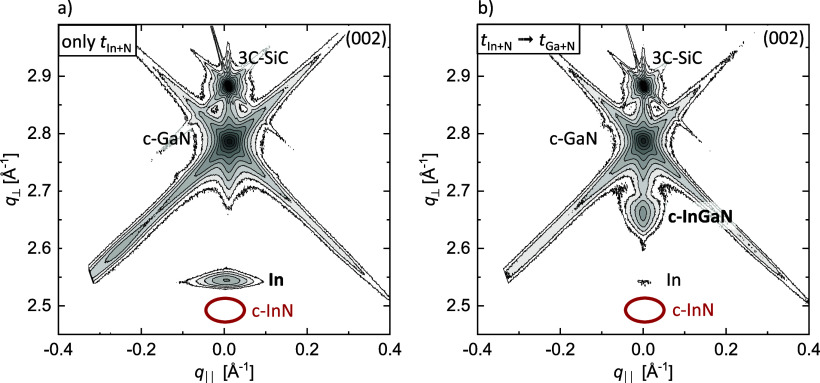
Reciprocal space maps centered around the symmetric (002) reflection of c-GaN after (a) a single indium deposition step (*t*_In+N_ = 180 s, BEP­(In) = 1.9 × 10^–7^ mbar) showing accumulated metallic indium. After (b) a subsequent Ga+N step, the RSM exhibits a c-InGaN reflection instead. In both cases, no c-InN is detected (*T*_growth_ = 615 °C).

The formation of c-InGaN occurs only when the growth sequence is extended by adding an additional Ga+N step. The corresponding RSM in Figure 2b shows a distinct c-InGaN reflection accompanied by only a minor amount of indium, as indicated by a clearly diminished peak intensity. Neither RSM shows any indication of a c-InN reflection. This implies that the chosen growth temperature suppresses InN formation. This experiment straightforwardly underscores the necessity of the GaN cycle following the deposition of indium to enable the formation of c-InGaN.

Time-of-flight secondary ion mass spectrometry (ToF-SIMS) and scanning transmission electron microscopy (STEM) spatially resolve c-InGaN formation. Figure 3a depicts the nominal layer sequence, i.e., assuming instantaneous growth of alloys from the supplied elements without ion exchange. The red layer represents the period of opening both indium and nitrogen shutters (*t*_In+N_ = 300 s). This layer is labeled as indium deposition, as inferred from the RHEED data. Tailored MME-grown c-GaN layers reveal the c-InGaN formation process: GaN_bottom_ and GaN_top_, with a nominal thickness of 30 nm, encapsulate the indium layer. Furthermore, two c-AlN layers spatially separate this GaN/InGaN/GaN stack from the c-GaN template and the surface. This allows for reliable determination of the MME-grown layers’ thicknesses.

**Figure 3:**
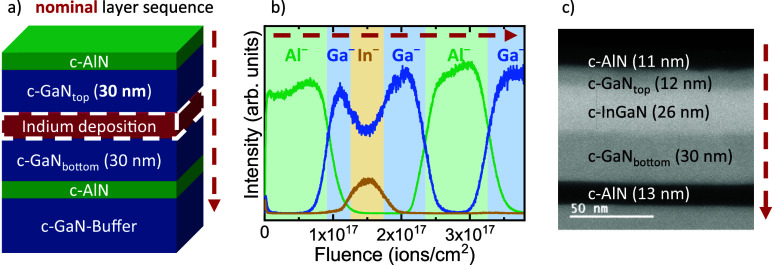
(a) Nominal layer sequence of a sample designed to spatially resolve the formation of c-InGaN (*T*_growth_ = 620 °C, *t*_In+N_ = 300 s, BEP­(In) = 1.9 × 10^–7^ mbar). (b) ToF-SIMS depth profile confirms the confinement of indium and reveals an asymmetric indium incorporation. (c) HAADF STEM precisely determines the layers’ thicknesses and confirms that indium is not incorporated in the bottom c-GaN layer.

Figure 3b shows a ToF-SIMS depth profile of the In^–^, Ga^–^, and Al^–^ intensities from the surface to the c-GaN template. The profile aligns with the nominal layer sequence in Figure 3a. It unambiguously demonstrates that indium is encapsulated by GaN and does not segregate to the surface. The coexistence of In^–^ and Ga^–^ in this layer suggests the formation of InGaN. XRD space maps confirm the presence of c-In*_x_* Ga_1–*x*_N with *x*(In) = 0.23 and show no indications of c-InN (see Figure S2). However, the fluence required to sputter through GaN_bottom_ and GaN_top_ during the SIMS depth profiles differs by a factor of 1.8. This implies that GaN_top_ is significantly thinner than GaN_bottom_, suggesting asymmetric indium incorporation.

HAADF-STEM imaging of a cross-section (Figure 3c) shows well-defined layers with clear interfaces and reveals the layer thicknesses. The measured layer thickness of 30 ± 1 nm for GaN_bottom_ exactly corresponds to the expectation, considering the growth rate and time. This outstanding agreement further evidences that indium does not incorporate into the underlying c-GaN layer. Comparing the amount of gallium in GaN_top_ + InGaN to that in GaN_bottom_ reveals whether the indium deposition affects gallium incorporation. The c-In_0.23_Ga_0.77_N layer is 26 ± 1 nm thick, whereas GaN_top_ has a thickness of only 12 ± 1 nm. Considering the layers’ thicknesses, the lattice constant of c-GaN *a*_GaN_ = 4.505 ± 0.010 Å, and the partially strained, out-of-plane lattice constant of c-In_0.23_Ga_0.77_N, *a*_InGaN, oop_ = 4.720 ± 0.010 Å, yields an estimate for the number of c-GaN lattice constants along the growth direction in each section, resulting in #*a*(GaN)_top+InGaN_ = 69.05 ± 4.00 and #*a*(GaN)_bottom_ = 66.59 ± 2.37. The amount of incorporated gallium is virtually identical in both sections. Consequently, the accumulated indium at the surface has no significant influence on the adsorption and incorporation of impinging gallium. Also, comparing the amount of incorporated indium for different *t*_In+N_ values indicates minor indium desorption during *t*_In+N_. This effect can be minimized by increasing the indium flux and shortening the *t*_In+N_.

The combined *ex situ* analysis by ToF-SIMS and STEM unambiguously shows that the accumulated indium forms a homogeneous c-InGaN layer when gallium and nitrogen are present. The indium lateral diffusion appears to be sufficiently fast to ensure steady indium distribution within the adlayer. Furthermore, the absence of indium in GaN_top_ implies that the indium reservoir is fully consumed by InGaN growth, which is important for quantum well fabrication.

The potential of metal-modulated growth for c-InGaN and self-assembled c-InGaN/GaN QWs highlights intrinsic parameter limitations such as a sufficiently high growth temperature and gallium flux. One major prerequisite for the formation of c-InGaN using the metal-modulated growth scheme is the accumulation of indium without the formation of c-InN. This has been achieved by choosing a sufficiently high growth temperature. Reducing *T*_growth_ below the InN decomposition temperature *T*_InN_ should result in InN formation. Figure 4a shows the RHEED pattern evolution when the gallium shutter is closed and the indium shutter is opened at *T*_growth_ < *T*_InN_. The streaky c-GaN pattern observed upon supplying indium does not blur, which would typically indicate indium accumulation. Instead, its transition to a spotty pattern suggests the formation of c-InN during *t*_In+N_. Correspondingly, Figure 4b presents the (−1–13) reciprocal space map of a sample grown at such *T*_growth_ < *T*_InN_. It clearly exhibits a distinct c-InN reflection but has no indication of any c-In*_x_* Ga_1–*x*_N. This transition from indium accumulation to c-InN formation occurs at a growth temperature of approximately 550 °C. In metal-modulated epitaxy, growing slightly above this transition temperature (*T*_growth_ ≥ *T*_InN_) yields an indium content of *x*(In) ≈ 0.37. Note that in conventional growth, the growth rate of c-InGaN increases at a similar temperature,^23^ supporting an increase in the stability of indium–nitrogen bonds.

**Figure 4:**
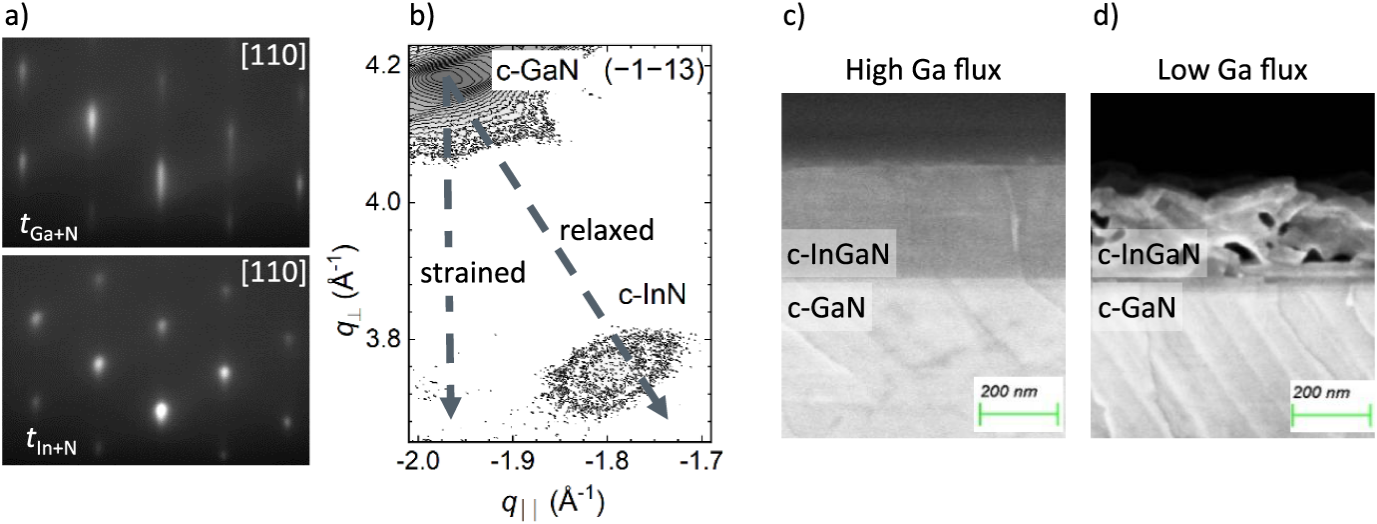
Intrinsic limitations of the alternating metal-modulated growth of c-InGaN. (a) RHEED and (b) a (−1–13) reciprocal space confirm that an insufficiently high growth temperature leads to c-InN formation during *t*_In+N_ (*T*_growth_ = 520 °C, *t*_In+N_ = 90 s, BEP­(In) = 2.9 × 10^–7^ mbar). Cross-sectional SEM images of MME-grown c-InGaN layers using a (c) high and (d) low Ga flux showing that sufficient Ga flux is required to achieve homogeneous layer quality (*T*_growth_ = 580 °C, *t*_In+N_ = 280 s, BEP­(In) = 1.9 × 10^–7^ mbar).

The rapid lateral distribution of indium requires a closed metal adlayer for enhanced diffusion mobility to obtain a homogeneous c-InGaN layer. Comparing two samples grown with two different gallium fluxes (BEP­(Ga,low) = 1.3 × 10^–7^ mbar and BEP­(Ga,high) = 2.2 × 10^–7^ mbar) reveals the contribution of gallium and indium toward maintaining the adlayer. The amount of supplied indium is sufficient to obtain a fully alloyed layer in both cases. Consequently, the indium reservoir is never fully consumed during *t*_Ga+N_. Figure 4c,d compares scanning electron microscopy (SEM) cross-section images of the c-InGaN layers grown using the different Ga fluxes. The high Ga flux (Figure 4c) yields a homogeneous c-In*_x_* Ga_1–*x*_N layer with a moderate indium content (*x*(In) = 0.24), while the low Ga flux (Figure 4d) results in a higher indium content (*x*(In) = 0.4). However, the latter features a rough and porous c-InGaN layer, even though the sample was grown under overall metal-rich conditions. This provides evidence that the majority of excess indium is localized in droplets that do not contribute to the adlayer. Lateral diffusion within the adlayer is reduced if the supplied gallium flux is insufficient, rendering the layer porous. Therefore, we estimate an upper limit of approximately 37% indium content for the metal-modulated growth scheme investigated in this work. Lower Ga fluxes can increase this content but are detrimental to the film quality of thicker c-InGaN layers.

## Conclusion

The growth mechanism for the formation of c-InGaN quantum wells in alternating metal-modulated molecular beam epitaxy is derived based on *in situ* RHEED measurements and *ex situ* characterization using HRXRD, ToF-SIMS, and STEM. These data reveal the prerequisites for the self-assembled c-InGaN formation. More specifically, XRD demonstrates the accumulation of indium without the formation of c-InN during *t*_In+N_. Additionally, ToF-SIMS depth profiling and STEM imaging show that the accumulated indium forms homogeneous and well-defined c-InGaN layers when subsequently supplying gallium and nitrogen. Further analysis identifies the need for a sufficiently high substrate temperature and gallium flux as intrinsic limitations of the metal-modulated growth. Nonetheless, estimated indium contents of up to 37% are viable using this approach, which encourages its implementation for red light-emitting devices. Correlating these findings with RHEED diagnostics during growth paves the way toward implementing AI and machine learning algorithms for automated growth of complex heterostructures for, e.g., quantum cascade applications.

## Supplementary Material



## Notes

Figure S1: RHEED pattern observed during the individual growth phases, Figure S2: reciprocal space map around the (−1–13) reflection of c-GaN revealing a strained c-In_0.23_Ga_0.77_N phase (PDF)
