# Supplementary material for: Mechanism of Self-Assembled Cubic InGaN/GaN Quantum Well Formation in Metal-Modulated Molecular Beam Epitaxy
Source: Cryst Growth Des. 2025 May 17;25(11):3793–9. doi: 10.1021/acs.cgd.5c00202 (PMC12142808; doi:10.1021/acs.cgd.5c00202)
Supplement: Supplementary file 1 [file cg5c00202_si_001.pdf]

Supporting Information

Mechanism of self-assembled cubic InGaN/GaN quantum well formation in metal-modulated molecular beam epitaxy

*Mario F. Zscherp[1], Silas A. Jentsch[1], Vitalii Lider[2], Matthew Chia[1,3], Andreas Beyer[2], Anja Henss[1], Donat J. As[4], Kerstin Volz[2], Sangam Chatterjee[1], Jörg Schörmann[1]**

[1] Institute of Experimental Physics I and Center for Materials Research, Justus Liebig University Giessen, Giessen, Germany

[2] Materials Science Center and Faculty of Physics, Philipps-University Marburg, Marburg, Germany

[3] Department of Physics, Cambridge University, Cambridge, United Kingdom

[4] Department of Physics, Paderborn University, Paderborn, Germany

*Jörg Schörmann: Institute of Experimental Physics I and Center for Materials Research, Justus Liebig University Giessen, Heinrich-Buff-Ring 16, D-35392 Giessen, Germany, Email: Joerg.Schoermann@exp1.physik.uni-giessen.de

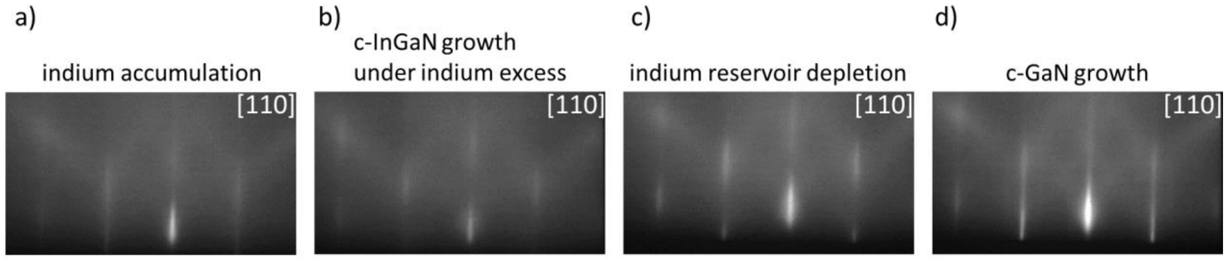

Figure S1. RHEED pattern observed during the individual growth phases. (a) Dim, but streaky pattern indicates metallic indium on c-GaN. (b) blurry pattern during c-InGaN growth under indium excess with (c) increasing intensity once the indium reservoir is consumed. (d) Clear and streaky pattern for the growth of c-GaN indicating a smooth surface.

During the metal-modulated growth of c-InGaN, four characteristic RHEED pattern (Figure S1) offer detailed insight on the growth mechanism. Figure S1a shows a streaky pattern with low intensity indicating metallic indium on the c-GaN surface. Growing c-InGaN under the cover of indium results in a dark and blurry pattern (Fig. S1b) due to a higher surface roughness. The increased intensity in Figure S1c corresponds to the consumption of the indium coverage. The growth of c-GaN yields a clear and streaky RHEED pattern (Fig. S1d) indicating a smooth surface.

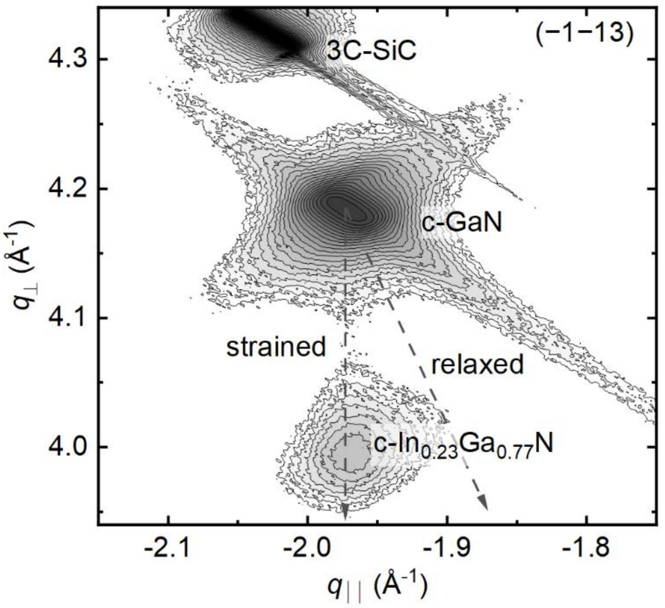

Figure S2. Reciprocal space map around the (−1−13) reflection of c-GaN for the sample presented in Figure 3, revealing a strained c-$In_{0.23}Ga_{0.77}N$ phase.

XRD confirms the formation of c-InGaN when growing a nominal layer sequence as shown in Figure 3a. Asymmetric reciprocal space maps are used to reliably determine the composition of strained ternary alloys. In Figure S2, such a space map around the (−1−13) reflection of c-GaN shows a peak corresponding to c-$In_xGa_{1-x}N$. By converting the reciprocal coordinates into an in-plane and an out-of-plane lattice parameter we determine an indium content x(In) = 0.23 and find that the c-InGaN layer is almost completely strained to the in-plane constant of c-GaN.
